# Ten Approaches That Improve Immunostaining: A Review of the Latest Advances for the Optimization of Immunofluorescence

**DOI:** 10.3390/ijms23031426

**Published:** 2022-01-26

**Authors:** Ricardo Piña, Alma I. Santos-Díaz, Erika Orta-Salazar, Azucena Ruth Aguilar-Vazquez, Carola A. Mantellero, Isabel Acosta-Galeana, Argel Estrada-Mondragon, Mara Prior-Gonzalez, Jadir Isai Martinez-Cruz, Abraham Rosas-Arellano

**Affiliations:** 1 Departamento de Biología, Facultad de Ciencias Básicas, Universidad Metropolitana de Ciencias de la Educación, Santiago 7760197, Chile; ricardo.pina@umce.cl; 2 Departamento de Fisiología, Biofísica y Neurociencias, Cinvestav del IPN, Ciudad de México 07360, Mexico; alma.santos@cinvestav.mx; 3 Departamento de Neurobiología del Desarrollo y Neurofisiología, Instituto de Neurobiología, Universidad Nacional Autónoma de México, Querétaro 76230, Mexico; erikaortabio@yahoo.com.mx (E.O.-S.); araguilar@comunidad.unam.mx (A.R.A.-V.); 4 Facultad de Salud y Ciencias Sociales, Universidad de Las Américas, Santiago 7500975, Chile; carola.mantellero@ubo.cl; 5 Departamento de Ciencias Químicas y Biológicas, Universidad Bernardo O’Higgins, Santiago 8370993, Chile; 6 División de Neurociencias, Instituto de Fisiología Celular, Universidad Nacional Autónoma de México, Ciudad de México 04510, Mexico; iacosta@ifc.unam.mx (I.A.-G.); mprior@ifc.unam.mx (M.P.-G.); jadirm@ifc.unam.mx (J.I.M.-C.); 7 Department of Biomedical and Clinical Sciences, Linköping University, 581 83 Linköping, Sweden; argel.estrada@liu.se; 8 Unidad de Imagenología, Instituto de Fisiología Celular, Universidad Nacional Autónoma de México, Ciudad de México 04510, Mexico

**Keywords:** immunolabeling, immunohistofluorescence, immunocytofluorescence, immunolocalization, antibody, signal-to-noise ratio, unmask epitopes

## Abstract

Immunostaining has emerged as one of the most common and valuable techniques that allow the localization of proteins at a quantitative level within cells and tissues using antibodies coupled to enzymes, fluorochromes, or colloidal nanogold particles. The application of fluorochromes during immunolabeling is referred to as immunofluorescence, a method coupled to widefield or confocal microscopy and extensively applied in basic research and clinical diagnosis. Notwithstanding, there are still disadvantages associated with the application of this technique due to technical challenges in the process, such as sample fixation, permeabilization, antibody incubation times, and fluid exchange, etc. These disadvantages call for continuous updates and improvements to the protocols extensively described in the literature. This review contributes to protocol optimization, outlining 10 current methods for improving sample processing in different stages of immunofluorescence, including a section with further recommendations. Additionally, we have extended our own antibody signal enhancer method, which was reported to significantly increase antibody signals and is useful for cervical cancer detection, to improve the signals of fluorochrome-conjugated staining reagents in fibrous tissues. In summary, this review is a valuable tool for experienced researchers and beginners when planning or troubleshooting the immunofluorescence assay.

## 1. Introduction

Immunostaining, also known as immunolabeling or immunolocalization, is one of the most widely used techniques in clinical diagnosis, and in both biological and histopathological research; this method is applied to describe the localization of protein populations, distributions, as well as abundance [1,2,3]. Remarkably, to date this method has prevailed, essentially preserving its initial methodological design [4]. In addition to being used as a routine laboratory work-up, the development of immunolocalization protocols itself is an area of deep research and upgrades [5,6,7,8,9]. Technically, to identify the direction in which this procedure will be applied, the addition of prefixes, such as cyto or histo, is used when this method describes antigenic sites within cells or tissues, respectively. The suffixes chemistry and fluorescence are also assigned when enzymes or fluorochromes are used for antibody labeling (Table 1) [2,4]. Enzymatic or fluorescence signals given by conjugated antibodies are subsequently coupled to bright-field or fluorescence microscopy, respectively. Otherwise, when nanogold-conjugated particles are used to label antibodies, this technique can be referred to as immunogold, immunocolloid, or immunoelectron microscopy, the latter being based on the use of scanning or transmission electron microscopy to visualize nanogold particles [10,11,12].

Regardless of the use of enzymes or fluorochromes, the immunolabeling assay is subdivided into two methods called direct or indirect. Direct immunostaining applies when only a primary antibody directly attaches to a label (conjugate antibody), whereas in the indirect method a labeled secondary antibody recognizes a non-conjugated primary antibody, which is designed to bind to its antigenic affinity site [13]. The direct method has several advantages over the indirect method. Firstly, it is an ideal option if the researchers have large amounts of conjugated primary antibody available; secondly, it is quick, and has a large signal-to-noise ratio due to a reduced number of non-specific sites to which secondary antibodies bind. Nevertheless, indirect immunostaining has its disadvantages, such as reduced sensitivity; hence, a large amount of antibody may be required to counteract it, decoupling the antibody and its labeling which reduces the immunosignal. Besides this, conjugated primary antibodies can be expensive. On the other hand, for the indirect method an unlabeled primary antibody is recognized by one or more conjugated secondary antibodies; these were designed to distinguish the antigenic sites of the species where the primary antibody was generated (i.e., anti-goat, anti-rabbit, and anti-mouse, etc.). The indirect method has several advantages, such as the primary antibody being stable, an enhanced signal given by the binding of one or more secondary antibodies to the primary antibody, and the low cost of secondary conjugated antibodies. Nonetheless, the indirect method has some disadvantages as well. It is a time-consuming procedure, displays a high background signal, and shows aberrant crosslinking, which is more frequent in the multiple labeling protocol [4].

Depending on the nature of the label attached to the antibody, these may also show additional advantages and disadvantages, for example, enzymatic tags show higher sensitivity and therefore less antibody is required to exhibit long-term staining; however, these show high background signals due to the spillover of the products of the enzymatic reaction, tissue and cell damage associated to the enzymatic reactions, less resolution of fine structures, and a poor variety of colorimetric markers, consequently limiting the possibility of multiple immunolabeling. On the other hand, fluorescent tags display high sensitivity and a large variety of fluorochromes are available, thus generating the possibility of multiple labeling in a single assay; remarkably, fine structures are well-resolved under fluorescent microscopes (widefield, confocal, etc.). As it was mentioned before, the fluorescence method can exhibit high background noise (this drawback might be a major issue in the indirect method), limited photostability, quenching (reduction of the emission of the fluorescence by the fluorochrome), and photobleaching (irreversible loss of the emission of fluorescence). Finally, the immunogold technique exhibits some advantages, such as high resolution, since it is coupled to scanning and transmission electron microscopy; however, it also has disadvantages, such as the short shelf-life of antibodies, difficulties in the performance of multiple immunolabeling, the fact that nanogold-conjugated antibodies are expensive, and because processing samples is time-consuming [4,14].

**Figure 1:**
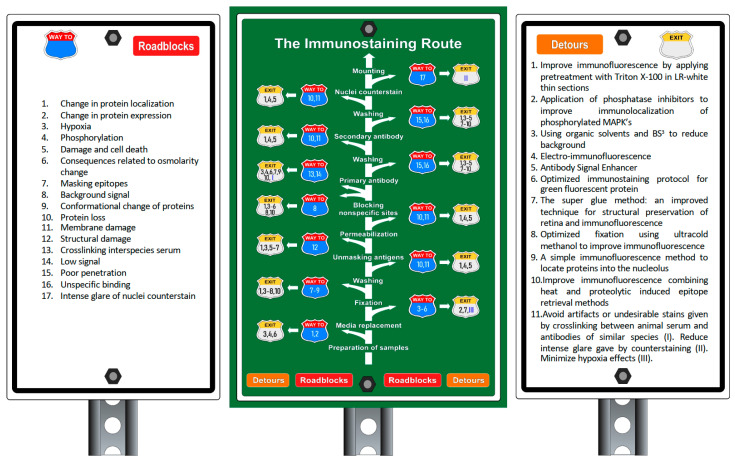
The immunostaining method and its common disadvantages through the metaphor of a highway: Vertical white arrow displays routine steps of the indirect immunolabeling method (The Immunostaining Route); after every stage some usual drawbacks (Roadblocks) related to each step are represented with numbers (Way to) and described in the road signs of roadblocks (**left**). Some of these technical disadvantages are tackled by the methodologies (Detours) presented in this review and represented with the corresponding number (Exit). Title of protocols in order of display are in road signs of detours (**right**). Roman numerals I, II, and III correspond to the section of additional tips (1, 7, and 9 respectively).

Theoretically, researchers always expect that at the end of the immunolocalization steps samples remain with minimal or null disturbances under the general procedure that consists of the following general points (The Immunostaining Route; Figure 1) [15,16,17]:

1. Sample preparation, related to the handling and maintenance of animals and cells under laboratory conditions;
2. Replacement of blood (for whole animals) or serum media (from cells or isolated tissues) by saline buffers;
3. Fixation, usually followed by a post-fixation step with an appropriate solution and temperature that vary depending on the tissue and organism type;
4. Antigen retrieval;
5. Permeabilization, sometimes as an independent step or carried out during the blocking stage, washing, or also applied during antibody incubations or nuclei counterstaining;
6. Blocking of non-specific sites. In this step, reagents are generally applied before the primary antibody incubation, and it often is also used as the antibody’s incubation buffer and/or as rinse solution besides the blocking buffer;
7. Primary antibody incubation. As mentioned above, primary antibodies are sometimes directly conjugated (direct method) or non-conjugated (indirect method);
8. Secondary antibody incubation (indirect method);
9. Washing (usually performed after fixation), antigen retrieval, and blocking incubation, as well as being followed by the incubation of primary and secondary antibodies;
10. Nuclei counterstaining, usually a complementary step to immunostaining.

### Drawbacks in Immunolabeling Methods

The drawbacks in every step of the immunolabeling methods are diverse (Figure 1, Roadblocks). Altogether or independently, these inconveniences could certainly lead to the misinterpretation of results.

1. Changes in protein localization as a consequence of: (a) protein alterations, (b) membrane damage, (c) loss or relocation of proteins, and (d) disruption of the native structure of tissues and cells;
2. Changes in protein expression: as above;
3. Hypoxia: (a) drastic decrease of oxygen levels;
4. Phosphorylation: (a) protein alterations, (b) biochemical changes;
5. Damage and cell death: (a) drastic decrease of oxygen levels, (b) loss of osmolarity, (c) biochemical changes, (d) loss of membrane surface tension, (e) membrane disruption (specially in cold fixation), and (f) disruption of the native structure of tissues and cells;
6. Consequences related to osmolarity changes: (a) biochemical changes, (b) loss of membrane surface tension;
7. Masking epitopes: (a) modifications of antigenic sites, (b) over-fixation;
8. Background signal: (a) cell injury and cell death, (b) modifications of antigenic sites, (c) low signal-to-noise ratio, (d) fluctuations of protein structure, (e) autofluorescence, (f) low antibody signal, (g) unspecific binding, and (h) low antibody binding;
9. Conformational changes of proteins: (a) protein alterations, (b) drastic decrease of oxygen levels, (c) biochemical changes, (d) cell injury and cell death, (e) modifications of antigenic sites, and (f) disruption of the native structure of tissues and cells;
10. Protein loss: (a) loss of membrane surface tension, (b) membrane disruption (especially in cold fixation), (c) disruption of the native structure of tissues and cells;
11. Membrane damage: (a) loss of membrane surface tension, (b) cell injury and cell death, and (c) disruption of the native structure of tissues and cells;
12. Structural damage: (a) loss of osmolarity, loss of membrane surface tension, (b) cell injury and cell death, (c) membrane disruption (specially in cold fixation), and (d) disruption of the native structure of tissues and cells;
13. Crosslinking interspecies serum: (a) use of sera and antibodies of similar species, (b) primary antibody raised in the same species as the sample;
14. Low signal: (a) cell injury and cell death, (b) modifications of antigenic sites, (c) fluctuations of protein structure, (d) autofluorescence, (e) low penetration, or (f) low antibody binding;
15. Poor penetration: (a) disruption of the native structure of tissues and cells, (b) poor permeabilization;
16. Unspecific binding: (a) cell injury and cell death, (b) modifications of antigenic sites, (c) fluctuations of protein structure, and (d) crosslinking;
17. Intense glare: (a) given by nuclear overstaining.

In summary, there are three general caveats in the method. Firstly, whether it is applied directly or indirectly; secondly, the types of labels used for antibodies; thirdly, the drawbacks associated with each step of the procedure. Immunolabeling protocols have been improved through the years, these improved protocols include improved avidin-biotin labeling to increase the signal-to-noise ratio [1,18]; the digestion with pepsin and trypsin to expose antigenic sites [19]; the utilization of microwave ovens to rescue epitopes [20]; applying biotinylated tyramide for higher sensitive immunolabeling [21]; reducing high background staining by using quenching reagents, such as copper sulfate or Sudan black B [6,22]; amplifying the peroxidase signal by adding a third antibody, an assay called the immunoenzyme-bridge technique [8]; and including a fourth antibody to increase even more of the peroxidase signal, a method known as the peroxidase-anti-peroxidase complex [5].

Improvements of the techniques have resulted in countless protocols involving a plethora of reagents and antibodies; each protocol assumes the use of specific concentrations of reagents and mixtures, as well as modifications of incubation times, with or without ranges of temperature [4,17]. Regardless of the variables, all adaptations in the immunolabeling method are designed to solve the same issues, namely antibody specificity, ultrastructural changes, and signal-to-noise ratio [9,23].

Likewise, several innovations have also been made for immunofluorescence, examples of these strategies include increasing antibody permeabilization [2,15,24,25]; remotion of aldehyde excess [15]; background reduction, avoiding the artifact’s increase of the signal-to-noise ratio, as well as antibody specificity [2,15]; epitope retrieval by proteolytic and/or heat treatments [24,26,27]; fixation improvements and organic degradation [28,29]; avoiding the dephosphorylation of proteins [30]; deep antibody penetration into “hidden” structures [31]; and enhancing antibody signals in an entire and well-preserved retina structure [32].

In the present review, we have collected and summarized 10 original proposals that have been shown to significantly improve immunofluorescence, highlighted by their innovative ingenuity. These approaches go hand in hand with a scientific curiosity based on a simple, but at the same time interesting design. We hope this review will facilitate the search and application of these alternative methods and help basic science and clinical researchers to improve routine immunofluorescence protocols. Each of the following procedures is accompanied by a short introduction restructured from the original manuscripts. The disadvantages that each method resolves are included in this section.

At last, but not least important, we extracted the core immunostaining improvements in these protocols and removed the original positive and negative controls to avoid confusion, because each control from original manuscripts was designed to prove the effectiveness of every improved method here presented; therefore, those looking to apply these methods must consider the use of its corresponding experimental control (knockout models, peptides, and no application of the primary antibody, etc.). Importantly, here we offer two approaches: (1) apply the chosen protocol step by step; or (2) an alternative fast protocol in which the user can adjust their standard protocol by specifically adding the key steps that improve the signal; scilicet, to include into your immunofluorescence procedure those steps that provide the improvements, applying them only for those situations when some antibodies do not allow protein detection, rather than changing the protocol completely. A similar addition of a key step has been previously tested in Flores-Maldonado et al. [2] for a triple immunofluorescence combining standard immunolabeling methods for two antibodies with the antibody signal enhancer (blocking and antibody buffers) for an antibody that did not generate any signal; this addition to the standard protocol produced an improvement of the immunolabeling process. Based on this scenario, we have highlighted strategic steps, named “key points”, that can be found at the end of each procedure, in order to add it to your protocol. We believe that including one or more of these key points in your routine protocol may help to quickly and clearly identify the most appropriate steps to add according to the specific needs in each situation. By the end, as a final step into each original procedure, we discuss the potential disadvantages which must be considered.

## 2. Methods to Improve Immunofluorescence

Methods to improve immunofluorescence that are presented as detours of Figure 1.

### 2.1. Improve Immunofluorescence by Applying Pretreatment with Triton X-100 in LR-White Thin Sections

#### 2.1.1. Drawbacks to Face

Disadvantages to face that are presented in (Figure 1): 7–12, and 16.

Antigen damage, loss of proteins, low signal, and poor penetration of antibodies are, apparently, inevitable consequences of processing samples for immunostaining [33]. Ghrebi et al. [34] created a modification for a technique looking to preserve antigens, increase antibody permeability, and reduce unspecific binding; they used mild fixation, partial dehydration, and embedding in LR-White [34]. LR-White is a low crosslinked hydrophilic acrylic resin, with low viscosity (10 cps), efficient for preserving antibody specificity in the immunogold technique [12,35]. This resin increases the signal-to-noise ratio, displaying high permeability to reagents and a suitable preservation of samples. Triton X-100 is one of the most routinely used agents to permeabilize biomembranes [36]. Pretreating with 0.2% Triton X-100 helps to improve immunofluorescence-staining intensity, as is mentioned in their manuscript. The authors also demonstrated that nuclear morphology, integrity of cell membranes, and cytoplasmic structures are consistently well preserved via analysis by scanning electron microscopy. It was tested on rat alveolar macrophages in vitro.

#### 2.1.2. Immunolabeling for Cell in Culture Embedded in LR-White and Pretreated with Triton X-100

1. Collect and centrifuge cell suspension for 5 min *;
2. Resuspend pellet in 5 mL of 0.1 M phosphate-buffered saline (PBS), pH 7.3;
3. Centrifuge again;
4. Wash cell pellets in PBS twice for 2 min;
5. Before fixation, always pretreat with 0.2% Triton X-100 (Fisher Scientific) for 2 min;
6. Fix with 4% formaldehyde (Fisher Scientific) and 0.2% glutaraldehyde (Sigma-Aldrich) for 15 min;
7. Wash with PBS three times for 5 min each;
8. Partially dehydrate cells with 30%, 50%, and 70% ethanol for 5 min each;
9. Gradually infiltrate cells with LR-White (Canemco) using a ratio of 2:1 LR-White over 70% ethanol for 1 h on a rotary device;
10. Infiltrate cells only with 100% LR-White twice for 1 h each with gentle agitation on a rotary device;
11. Cold cure cells:
  a. Cool an accelerator to 0 °C and mix with LR-White resin;
  b. Place samples in gauge gelatin capsules (Canemco);
  c. Filled with LR-White-accelerator mixture.
12. Place the sample-block in a refrigerator and leave to polymerize for 3 h at 0 °C;
13. Cut sections using an ultramicrotome, transfer to poly-L-lysine pre-coated glass slides (Electron Microscopy Sciences) and leave to dry overnight;
14. Block non-specific sites for 20 min in 3% bovine serum albumin (Sigma-Aldrich), 0.1% glycine (Electron Microscopy Sciences), 0.2% Tween 20 (Fisher Scientific), and 5% normal goat serum (Sigma Aldrich);
15. Add primary antibody in blocking solution and incubate for 1 h;
16. Rinse five times for 2 min each in PBS and block again for 20 min;
17. Incubate with secondary antibody for 1 h;
18. Rinse samples five times for 2 min in PBS and mount with Prolong antifade (Molecular Probes).

#### 2.1.3. Key Points 

Consider adding steps 5–13 to your immunofluorescence protocol.

#### 2.1.4. Disadvantages

(a) High cost of LR-White;
(b) This technique can only be used when the antigen is resistant to 0.2% of Triton X-100 and;
(c) Long-term Triton X-100 permeabilization induces cell membrane damage, as such do not apply for long periods at 0.2% or upper concentrations because even short times may increase fluorescence intensity, as has been previously reported when Triton X-100 viscosity is higher [37].

Note: * Often for LR-White procedure a few cells are enough.

### 2.2. Application of Phosphatase Inhibitors to Improve Immunolocalization of Phosphorylated MAPKs

#### 2.2.1. Drawbacks to Face

Disadvantages to face that are presented in (Figure 1): 4 and 6.

Phosphorylation of proteins in one or more residues induces its full activation which triggers gene expression, cell survival, proliferation, differentiation, and cell death [38], as in mitogen-activated protein kinases (MAPKs). The MAPKs are differentially phosphorylated displaying a specific function that allows for the distinguishing of each MAPK depending on its physiological role. Abnormal conversions from the phosphorylated (active) to dephosphorylated (inactive) state of the MAPKs or their regulators are of vital importance, as they can contribute to physiological processes. This may lead to the onset of pathologies, for example, Alzheimer’s and Parkinson’s diseases, or different types of cancer. Mammone et al. [30] indicated that the cardial perfusion procedure induces the dephosphorylation of proteins.

In the animal perfusion step, some drawbacks may be present, since the procedure produces hypothermia, anoxia, and vascular compromise, triggering disruption of homeostasis and causing the progressive decline of cellular activity, which induces variations in the levels of phosphorylated MAPKs. Typical phosphorylation of proteins occurs between 13–200 s [39], whereas dephosphorylation occurs in less than 1 min [40]. Taking all this into account, it is essential to stabilize MAPK phosphorylation state-specific molecules, avoiding its dephosphorylation promptly considering the slow ratio of penetration of fixation solutions (based on formaldehyde 1 mm/h) [41]. Freeze fixation has been reported to be a solution to prevent the dephosphorylation of proteins; however, it induces membrane disruption, structural changes, and cell death. As a simple alternative, the authors preserved phosphorylated protein levels before fixation by perfusing saline buffer mixed with a cocktail of tissue-permeable protein phosphatase inhibitors. This method was tested in the rat optic nerve and retina.

#### 2.2.2. Tissue Preparation

1. Perfuse transcardially using sterile physiological saline containing 1% (*v*/*v*) phosphatase inhibitor cocktail 2 (sodium orthovanadate, sodium molybdate, sodium tartrate, and imidazole; Sigma-Aldrich) and 0.01 M sodium fluoride;
2. Proceed with fixative Davidson’s solution (formaldehyde, absolute ethanol, glacial acetic acid, and water (use the deionized or similar water that you use for your routine buffers); 2:3:1:3; this solution has been reported to reduce dephosphorylation of MAPK);
3. Extract tissue and post-fix for 24 h by immersing in Davidson’s solution;
4. Transfer to 70% (*v*/*v*) ethanol;
5. Embed sections in paraffin;
6. Cut 5 μm serial slices using a rotary microtome;
7. Deparaffinize sections with xylene followed by 100% ethanol;
8. Microwave for 10 min in boiling sodium citrate buffer (10 mM; pH 6.0) (antigen retrieval). For power of microwave, you can review protocol 2.6, Step 7, for frozen sections and Protocols 2.6 and 2.10, Steps 4 and 2, respectively, for paraffin-embedded sections;
9. Block non-specific sites in PBS (137 mM NaCl, 5.4 mM KCl, 1.28 mM NaH_2_PO_4_, 7 mM Na_2_HPO_4_; pH 7.4) with 3% (*v*/*v*) normal horse serum (PBS-NHS).

#### 2.2.3. Immunofluorescence

##### Two-Step Method

1. Incubate overnight at room temperature (RT) in primary antibody diluted in PBS-NHS;
2. For secondary antibody in Alexa Fluor^®^ PBS-NHS conjugate for 30 min;
3. Nuclear counterstain using 500 ng/mL of 4′,6-diamidino-2-phenylindole (DAPI) in PBS;
4. Mount using fluorescence-preserving medium (Dako).

##### Three-Step Method

**Figure 2:**
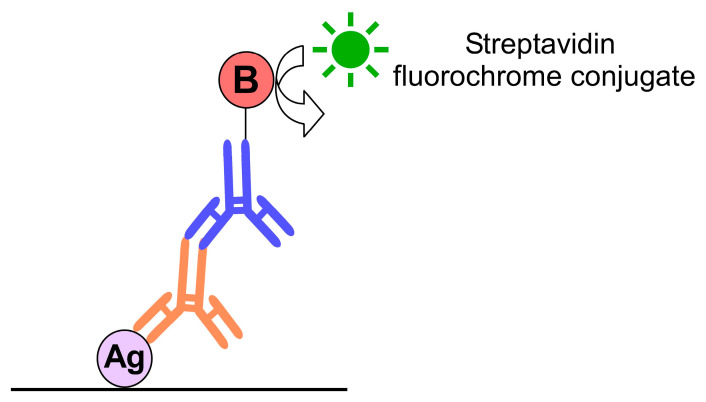
Three step immunofluorescence method: The first antibody (orange) is binding to an epitope (Ag). In turn, primary antibody is recognized by a secondary antiserum (blue). This antibody is coupled to biotin (B), and this molecule can be observed under microscope by the binding of streptavidin-conjugated fluorochrome complex.

Three-step method is shown in Figure 2.

5. Primary antibody in PBS-NHS overnight incubation at RT;
6. Biotinylated secondary antibody in PBS-NHS, incubation for 30 min;
7. Streptavidin-conjugated fluorochrome incubation for 1 h;
8. Counterstain with DAPI and mount as above.

#### 2.2.4. Key Points

Consider adding steps 1–3 of the tissue preparation to your immunofluorescence protocol.

#### 2.2.5. Disadvantages

(a) The relatively high cost of phosphatase inhibitors;
(b) Despite phosphorylated protein stabilization, this does not lead to antibody signal enhancement.

### 2.3. Using Organic Solvents and BS^3^ to Reduce Background

#### 2.3.1. Drawbacks to Face 

Disadvantages to face that are presented in (Figure 1): 1, 2, 7–9, 12, 14, and 16.

**Figure 3:**
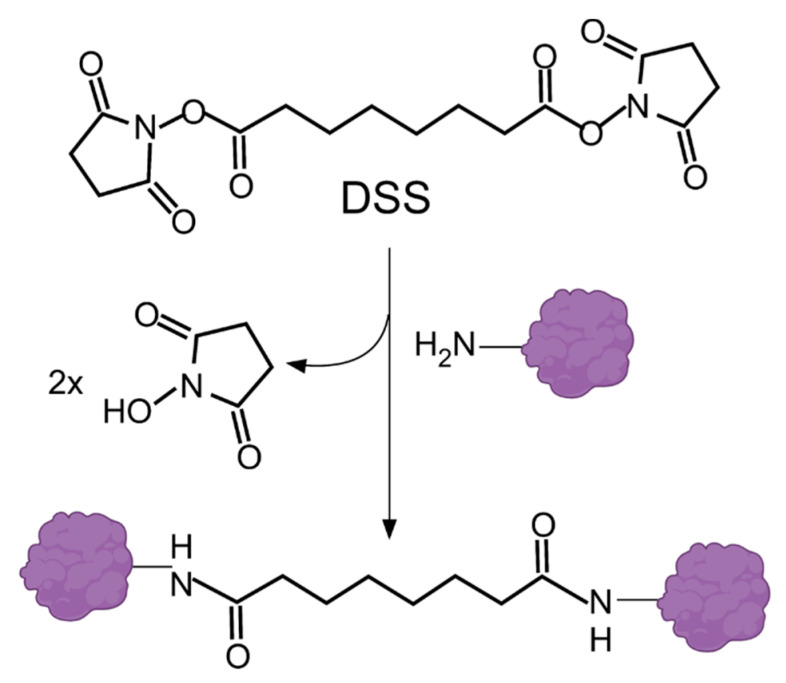
BS^3^ (bis(sulfosuccinimidyl)suberate) crosslinking: Linear chemical structure (**upper**); crosslinking reaction: the amino groups of proteins react with the N-hydroxysuccinimide (NHS) residues (**middle**); forming an amide bond (**lower**). Protein was adapted from “Icon Pack”, by BioRender.com (2021). Retrieved from https://app.biorender.com/biorender-templates. Accessed on 23 November 2021. Abbreviation: BS^3^ (Sulfo-DSS).

Bhattacharyya et al. [29] designed an immunolabeling technique to tackle the drawbacks generated by formaldehyde fixation, which often result in a loss of information due to epitope masking, low signal-to-noise ratio, conformational changes, and a loss of proteins, as well as alterations of cytoarchitecture induced by rinsing steps [42]. As has been reported, an alternative fixation with organic solvents, such as acetone or methanol, increases the definition of structures; however, results are not quite satisfactory because solvents modify cellular architecture during washing or fluid exchange. The protocol preserves intracellular sites by dehydrating cultured cells with cold organic solvents and then rehydrating them in fixative solution using a homobifunctional amine-reactive crosslinker, named as BS^3^ (bis(sulfosuccinimidyl)suberate), which is an amine-to-amine crosslinker and water-soluble compound able to bind to amine groups of proteins and antibodies—as mentioned by the authors. This crosslinked binding reaction stabilizes cellular architecture during rinses without compromising antibody binding (Figure 3). The authors tested their method on rat kidney cells.

#### 2.3.2. Organic Solvent Treatment and Crosslinking

1. Remove as much culture medium as possible and transfer the coverslip immediately (from 37 to −20 °C as quickly as possible, >5 s) into 25 mL of cold organic solvent (acetone or methanol) at −20 °C and leave for 5 min;
2. Remove a coverslip from the organic solvent (acetone or methanol) using forceps and dry vertically in a culture hood until the solvent has completely evaporated (results are better if the solvent is dried quickly);
3. Previously, prepare diluted bis(sulfosuccinimidyl)suberate (BS^3^) by adding 10 µL of 10 mM BS^3^ to 990 µL PBS, add to PBS 0.1% n-octyl-β-D-glucopyranoside (also known as octyl glucoside [OG]), a nonionic dialyzable detergent that reduces background signal. Incubate samples for 30 min at RT in diluted BS^3^ crosslinker in a humidified chamber. If the BS^3^ stain is poor, gentle rehydrate tissue in PBS;
4. Remove BS^3^ carefully, wash it three times with PBS, and then gently wash the buffer;
5. Incubate with ethylenediamine (100 mM) for 15 min at RT to quench unreacted BS^3^;
6. Centrifuge blocking buffer (to 10 mL PBS add 0.1 g non-fat powdered milk, 222 μL 45% cold water fish skin gelatin, and 0.1 mL normal horse serum) for 15 min at 2000 g. Transfer supernatant to a new tube and use this blocking buffer from now on.

#### 2.3.3. Antibody Incubations and Mounting

1. Use the centrifuged blocking buffer to block non-specific binding sites. Incubate for 1 h at RT;
2. Carefully aspirate or blot the blocking buffer. Incubate with primary antibody solution (blocking buffer plus antibody) for 30–60 min at RT;
3. Wash gently with blocking buffer eight times;
4. Incubate with secondary antibody solution for 30 min at RT;
5. Wash with blocking buffer 10 times;
6. Mount samples (for homemade mounting medium 90 mL glycerol, add 10 mL of 10X PBS, pH 9 with 0.5 M Na_2_CO_3_) and seal the edges with clear nail polish.

#### 2.3.4. Key Points

Consider adding steps 1–5 of organic solvent treatment and crosslinking to your immunofluorescence protocol.

#### 2.3.5. Disadvantages

(a) Fast dehydration with organic solvents could produce abrupt osmolarity changes and alterations of surface tension.

### 2.4. Electro-Immunofluorescence

#### 2.4.1. Drawbacks to Face 

Disadvantages to face that are presented in (Figure 1), 1, 2, 8–10, and 14–16.

Looking for a reliable description of protein populations and their representative distributions by immunolocalization, Liu and Kao [25] tackled the inconveniences of the loss of proteins and poor antibody penetration, both of which can occur as usual drawbacks of many if not all steps of the immunolabeling process (Figure 1). To overcome these problems, they used, on the one hand, a whole-mount tissue with the aim of reducing the loss of proteins, and on the other, they promoted antibody penetration by applying electrical currents using an electrophoresis chamber to drive fluorescent-conjugated antibodies and staining reagents deeply into the thick and dense tissue. The combination of both improvements also demonstrated a decrease in background signal noise by reducing non-specific antibody binding. They called their new and innovative technique electro-immunofluorescence staining, which is useful for examining antigen distribution in thick tissue, allowing the penetration of antibodies beyond that reported by the conventional method that ranges between 8–9 µm [43]. Electro-immunofluorescence was performed by applying a direct immunostaining method coupled to laser scanning microscopy. The authors tested this technique on a mouse cornea, which has an average thickness of 120 μm.

#### 2.4.2. Methods

**Figure 4:**
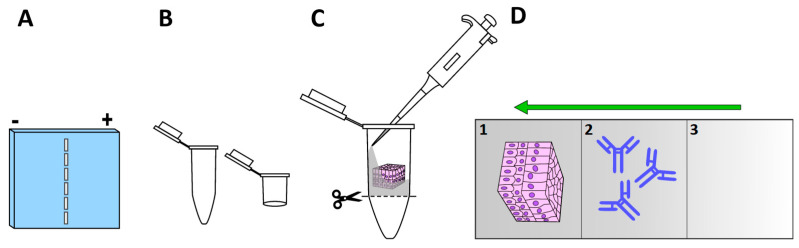
Key components of immuno-electrophoresis method: (**A**) 1% agarose gel. Loading wells must be positioned at the center to facilitate the detection of net charges of the antibodies and reagents; (**B**) suggested microtubes to adapt a tissue–antibody column; (**C**) place fresh 1% agarose (50–60 °C) in the bottom of selection tube, wait for polymerization, place the tissue in the proper orientation (layer of interest must go face up) over-polymerize agarose and fill gently with agarose and Triton X-100 in TGB as indicated in Step 1 of immunoelectrophoresis, then cut the plastic base of the tube and start to construct the column as indicated in the protocol; (**D**) column layers (1), tissue layer (2), antibody or reagent fluorochrome-conjugated layer (3), cover layer. Green arrow indicates the direction of migration. The green arrow indicates the direction of migration, which will depend on the net charge determined.

Determination of net charges of fluorescent reagents: to determine net charges of conjugated primary antibodies (the authors used anti-keratocan, anti-β-tubulin, and anti-FAK, all conjugated with Alexa Fluor^®^ 555) or staining reagents (DAPI and phalloidin):

1. Load IgG conjugates (around 150 kDa) and reagents onto a 1% agarose gel (Figure 4A) in Tris-glycine buffer (25 mM Tris and 250 mM glycine/HCl) [TGB]), pH 7.4;
2. Electrophorese at 10 mA for 4–6 h;
3. Observation using a UV stereoscopic microscope or gel-documentation system equipped with a fluorescent detector. Fluorescent reagents that migrate towards the positive pole are negatively charged, and vice versa.

#### 2.4.3. Tissue Preparation

1. Dissect tissue, fix, and post-fix by immersing in 0.1 M phosphate buffer containing 4% paraformaldehyde and 0.2% glutaraldehyde (this mix of fixatives aims to reduce loss of cytoplasmatic proteins during electrophoresis), pH 7.4, at 4 °C overnight;
2. Wash tissues with TGB;
3. Incubate with 0.1% Triton X-100 in TGB for 1 h;
4. Preparation of column for samples. We recommended hinged plastic containers (Electron Microscopy Sciences #72617-09 or #72617-11) (Figure 4B), or as an alternative, an Eppendorf microtube according to the dimensions of the tissue. The base of the microtube is removed to permit free exchange of TGB within the tube. The microtube containing the sample can be fixed inside an electrophoresis chamber adapting a plastic strip from an Eppendorf tube rack.

#### 2.4.4. Immunoelectrophoresis (Direct Immunofluorescence Method)

1. Layer 1: Use the bottom of vial to make layer 1 (if your tissue is polarized place the layer of interest facing up (Figure 4C)), prepare 1% agarose, and 0.05% Triton X-100 in TGB (heat and mix until 50–60 °C, and pour carefully into the vial containing the sample). Wait for polymerization;
2. Layer 2: Place an aliquot of 100 μL of 0.1–1 μg (1–10 μg per mL/sample) of primary conjugated antibody in 0.5% agarose (heat and place as point 1) on top of the specimen;
3. Layer 3: Seal antibody layer with 100 μL of TGB with 2% agarose (heat and place as point 1);
4. Cut the base of the vial or microtube to permit free diffusion of buffer and currents into the column;
5. In summary, the column should have three layers (Figure 4D);
6. Place the column in the appropriate direction according to the net charge of the staining reagents or conjugated primary antibodies;
7. Fill the electrophoresis chamber with 1X TGB;
8. Electrophorese for 10–24 h at 4–10 mA;
9. Extract tissues, mount the sample, and observe under a laser scanning microscope.

#### 2.4.5. Key Points

Consider adding steps 1–3 of net charges, 1 and 3 of tissue preparation, and steps 1–4 and 6–8 of immunoelectrophoresis to your immunofluorescence protocol.

#### 2.4.6. Disadvantages

(a) Antibodies will migrate only if they have a different isoelectric point than the TGB used in electrophoresis;
(b) This technique has not been tested for indirect immunolabeling or double and triple immunostaining. If you are looking to apply the two latter methods, the authors suggest the use of antibodies with identical isoelectric points and high binding affinity.

Note: In theory, you should not use combinations of reagents with different charges in the same sample. In the study, Alexa Fluor^®^ 555-conjugated antibodies showed different net charges (positive and negative), whereas DAPI and phalloidin have similar positive charges.

### 2.5. Antibody Signal Enhancer 

#### 2.5.1. Drawbacks to Face

Disadvantages to face that are presented in (Figure 1): 7–9, 11, 12, 15, and 16.

**Figure 5:**
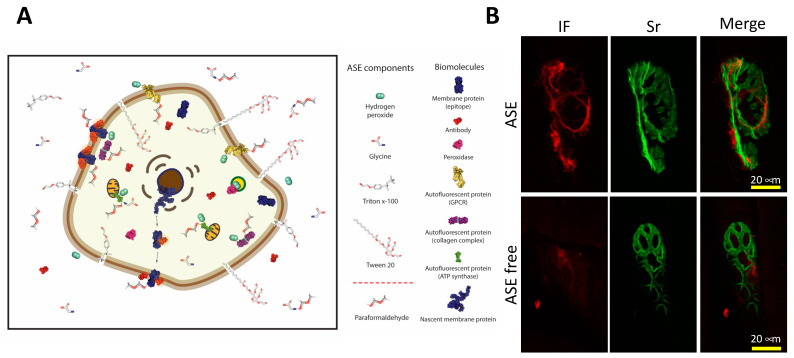
(**A**) Scheme of the effect of the antibody signal enhancer (ASE) over a eukaryotic cell. ASE solution components are indicated. Together, nonionic detergents Triton X-100 and Tween 20 poke holes in the membrane (enhanced permeabilization), and then antibodies and the rest of the ASE components (glycine and hydrogen peroxide) can get inside the cell. Frequently, some cell proteins are over-fixed, typically by paraformaldehyde or other aldehyde-fixing agents, and this excess is chelated by glycine molecule amino groups, favoring the binding of the antibody to the corresponding epitopes. Furthermore, hydrogen peroxide at micromolar concentrations successfully reduces autofluorescence without the quenching of the specific fluorescence or enzymatic signal, enhancing the specific signals, and reducing background noise from autofluorescence proteins or endogenous peroxidase from different cell compartments, such as membranes (e.g., GPCRs), cytoplasms (e.g., collagen complex) or organelles (e.g., lysosome, peroxisome). ASE can also enhance the signal of antibody-bound cytoplasmic proteins in transit from the nucleus or endoplasmic reticulum into their specific cell compartments previously permeabilized by both detergents (Triton X-100 and Tween 20); (**B**) Representative micrographs of *Bicep femoris* of male Wistar rat neuromuscular junction displaying immunofluorescence labeling (IF) and a staining reagent (Sr). In green is the postsynaptic nicotinic acetylcholine receptor (stained with α-bungarotoxin-Alexa Fluor^®^ 488 conjugate). In red label, given by secondary antibody Alexa Fluor^®^ 546 conjugate, is a representative immunofluorescence for S100 β-AF546 in perisynaptic Schwann cells. The immunostaining of *Bicep femoris* was performed under two different conditions using ASE method (upper), and using 2% BSA/0.2% Triton X-100 blocking solution as antibody incubation buffer (ASE free). The increase of signal using the ASE treatment was 3.339 for immunofluorescence, whereas 1.857 was the increase for fluorochrome-conjugated staining reagent. Intensity values were obtained using ZEN 2.6 blue edition Zeiss software (See Supplementary Materials).

Rosas-Arellano et al. [15] designed an antibody signal enhancer (ASE) to counteract the undesirable effects from over-fixation, poor antibody penetration, structural damage, and non-specific binding. ASE is an easy to prepare, simple, and inexpensive option that produces specific binding, and does not produce artifacts [2]. This antibody enhancer consists of two solutions: an ASE blocking buffer and an ASE antibody incubation buffer; both contain glycine to block unreacted aldehydes that produce background signals; low concentrations of H_2_O_2_, needed to quench autofluorescence by some fluorescent biomolecules [44]; and Tween 20 (nonionic detergent, surface tension reducer) and Triton X-100 (nonionic detergent penetration enhancer), that contribute to synergize cell membrane permeabilization (Figure 5A). The ASE method functions well for immunohistochemistry, immunofluorescence, and immunogold. In addition, in this review article, we demonstrated the use of ASE to increase fluorescent-conjugated reagents in fibrous tissue (Figure 5B and Supplementary Information), besides fluorescent-conjugated antibodies, as was previously demonstrated [2,15]. Interestingly, Flores-Maldonado et al., 2020, showed that ASE improved staining for several antibodies in various immunolabeling techniques for tissues and cells, including an enhanced detection of human cervical cancer in comparison with conventional clinical tests. ASE antibody incubation buffer is preferably, but not exclusively, applicable for non-fluorochrome-conjugated antibodies, because it contains H_2_O_2_, and this reagent could quench fluorescence—it breaks down when exposed to variation in temperature—therefore, it is recommended to always use fresh preparations of both enhancer solutions and to store the H_2_O_2_ reagent at 4 °C. The ASE method has been tested in Madin–Darby canine kidney cells, tissues of mice, rats, non-human primates, and human cervical cancer samples.

#### 2.5.2. Tissue Preparation

1. Transcardial perfusion of mice (3 min) by gravity using 155 mM NaCl, 1 mM MgCl_2_, 3 mM KCl, 3 mM NaH_2_PO_4_, 5 mM 4-(2-hydroxyethyl)-1-piperazineethanesulfonic acid (HEPES) pH 7.4, and 1 mM heparin sodium salt dissolved in perfusion solution, added immediately before perfusion into the perfusion system, and if possible, proximal to the animal;
2. Transcardial perfusion with fixative solution (30 mM HEPES, 100 mM NaCl, 4% paraformaldehyde, and 0.5% glutaraldehyde, pH 7.4) for 30 min;
3. Impregnate for 20 min (do not move the animal after perfusion);
4. Remove tissue and place in fresh fixation solution every 12 h for 2 days at 4 °C;
5. On the third day, perform final post-fixation in a fresh fixative solution for 2 h at RT.

#### 2.5.3. Immunohistofluorescence

1. Cryoprotect using sucrose gradients; 10% overnight, 20%, and 30% 24 h each, or until tissue precipitates to the bottom of the vial at 4 °C;
2. Embed in Tissue-Tek OCT compound (Sakura^®^ Finetek) and store at −20 °C for immediate use or at −80 °C for months;
3. Obtain 30 μm sections using a cryostat;
4. Wash in PBS with 0.5% Tween 20 twice for 3 min;
5. Block non-specific sites for 30 min using ASE blocking solution (2% donkey serum, 50 mM glycine, 0.05% Tween 20, 0.1% Triton X-100, and 0.01% bovine serum albumin [BSA] diluted in PBS);
6. Incubate in primary antibody diluted in ASE incubation solution (10 mM glycine, 0.05% Tween 20, 0.1% Triton X-100, and 0.1% H_2_O_2_ in PBS) overnight at 4 °C;
7. Rinse in PBS with 0.5% Tween 20;
8. Incubate with secondary antibody diluted in PBS plus 0.1% Tween 20 for 12 h at 4 °C;
9. Counterstain nuclei with Hoechst 33342 (Sigma-Aldrich) and mount with DAKO fluorescence mounting medium (DAKO).

#### 2.5.4. Immunocytofluorescence

1. Wash cells with PBS;
2. Place in methanol at −20 °C for 10 min for fixation and permeabilization;
3. Rehydrate at RT with PBS during 10 min;
4. Block non-specific sites using ASE blocking solution (see immunohistofluorescence);
5. Primary antibody incubation for 1 h at RT in ASE incubation solution (see immunohistofluorescence);
6. Rinse thrice with PBS;
7. Incubate in Secondary antibody diluted in 0.1% Tween 20 in PBS for 30 min at RT;
8. Wash thrice with PBS and twice with deionized water;
9. Mount using VectaShield mounting medium.

#### 2.5.5. Key Points

Consider adding steps 5 and 6 of immunohistofluorescence, 4 and 5 of immnunocytofluorescence to your immunofluorescence protocol. 

#### 2.5.6. Disadvantages

(a) Take care to not increase more than 0.6% H_2_O_2_ [44] when using with fluorescence-conjugated antibodies or reagents, hydrogen peroxidase can quench or bleach the fluorescence;
(b) ASE must always be prepared as a fresh solution.

### 2.6. Optimized Immunostaining Protocol for Green Fluorescent Protein

#### 2.6.1. Drawbacks to Face

Disadvantages to face that are presented in (Figure 1), 2, 7–9, 12, and 14.

**Figure 6:**
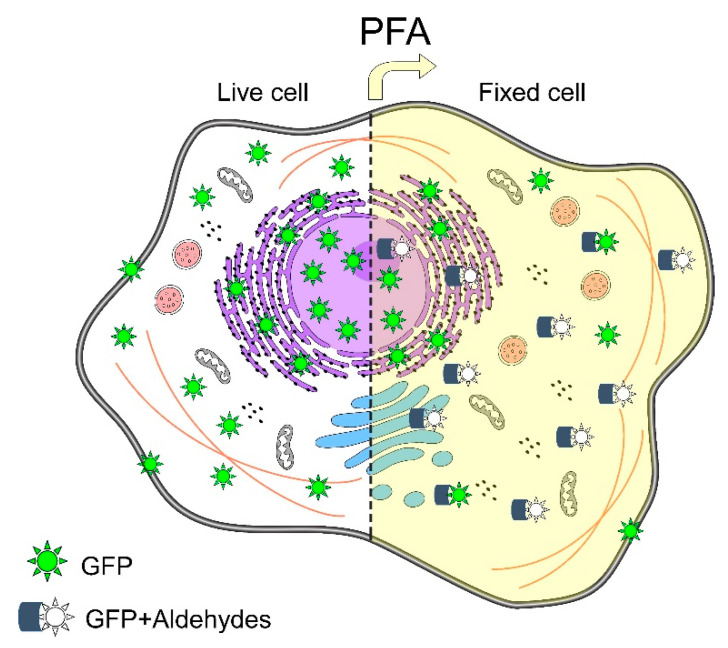
Schematic representation of green fluorescent protein (GFP)-fluorescence emission in a eukaryotic cell: In situ environment, the cellular spread of GFP-emitting fluorescence (**left**). GFP under chemical fixation with paraformaldehyde (PFA); in some PFA–GFP complexes, a conformational change of GFP is induced, and as consequence GFP remains non-fluorescent (**right**). Based on Pereira et al. [47].

Zaglia et al. [24] developed a protocol for the immunodetection of green fluorescent protein (GFP). GFP emerged to be an invaluable tool to study biological pathways and intracellular signaling; the success of the use of this fluorescent protein depends on its imperturbable structure and the subsequent capability to emit fluorescence [45,46]. Alterations in the structural integrity of fluorescent proteins may compromise its emission of fluorescence; GFP requires strong fixation to preserve its conformation; especially when GFP is located deeply within the tissue a long-term fixation is required. However, it produces autofluorescence, protein crosslinking, and epitope masking, leading to protein mislocalization due to the absence of fluorescence (Figure 6) [47,48]. The authors used their improved protocol by applying microwaves for the unmasking of epitopes, and 1% Triton X-100 for permeabilization to visualize deep intracellular and cell-surface GFP signals in human, rat, and mouse heart samples. They observed a significant improvement in the preservation of GFP-native fluorescence, antigenicity, and tissue morphology.

#### 2.6.2. Protocol Applying Transcardial Fixation

1. Perfuse with PBS 50 mL (rats) or 20 mL (mice) using a peristaltic pump at a rate of 200 mL/h (rat) or 75–100 mL/h (mouse);
2. Perfuse with 150 mL or 30 mL of 4% paraformaldehyde (Sigma Aldrich) for rats and mice, respectively;
3. Rinse in PBS and cryoprotect using a sucrose gradient (5% sucrose, 15 min; 15% sucrose, 30 min; sucrose 30% overnight at 4 °C);
4. Cryo-embed in OCT (Kaltek) and fast freeze in liquid nitrogen;
5. Obtain 10 μm-thick sections using a cryostat;
6. Microwave irradiation: Fill Coplin jars with 200 mL of 1 mM ethylenediaminetetraacetic acid (EDTA) pH 8.0, and incubate glass slides. Note: always use the same quantity of coverslips for each Coplin jar. Use an equal volume of buffer and coverslips—use empty coverslips if necessary. Use identical jars and place them in the same position in the microwave oven;
7. For frozen sections, perform three consecutive heat–cool cycles for 5 min at 240 W, then cool slowly to 37 °C. The bathing buffer should be between 65 and 68 °C at the end of the heating cycle;
8. Permeabilize and incubate with primary antibody using PBS with 1% BSA and 1% Triton X-100 for 12 h at 4 °C and then 2 h at 37 °C;
9. Incubate with secondary antibody at 37 °C for 2 h using the antibody incubation buffer from the previous step.

#### 2.6.3. Protocol for Long-Term Fixation by Immersion

1. Fix samples with formalin (interval 3 to 6 weeks), and then embed in paraffin (Perintelsint Rvg/2 Kaltek srl);
2. Obtain microtome sections (3 µm thick) on glass coverslips (Superfrost plus);
3. Deparaffinize cut sections;
4. Perform antigen retrieval by immersing the sections in 1 mM EDTA pH 8.0 and irradiating at 750 W using a microwave oven. Boil in a bathing buffer for 45 s and then cool slowly to 45 °C. Repeat the cycle eight times;
5. Permeabilize sections in PBS supplemented with 1% BSA and 10% Triton X-100 for 2 h at 37 °C;
6. Perform extended permeabilization overnight at 4 °C during primary antibody incubation (see Step 7 of transcardial perfusion);
7. Perform secondary antibody incubation diluted in the same buffer of primary antibody.

Note: 4% paraformaldehyde strongly affects GFP immunoreactivity; however, it can partially be reconstituted by the microwave treatment described above. The efficiency can be increased during the permeabilization step and antibody incubation using 10% and 1% Triton X-100, respectively.

#### 2.6.4. Key Points

Consider adding steps 6–9 of perfusion and 4–7 of immersion to your immunofluorescence protocol.

#### 2.6.5. Disadvantages

(a) For optimal parameter control we recommend a specialized laboratory microwave;
(b) High concentrations of Triton X-100 induce membrane damage and autofluorescence [37] and;
(c) excessive paraformaldehyde fixation increases free aldehydes and crosslinking.

### 2.7. The Super Glue Method: An Improved Technique for Structural Preservation of Retina and Immunofluorescence

#### 2.7.1. Drawbacks to Face

Disadvantages to face that are presented in (Figure 1): 6, 12, 14, and 15.

Structures associated with the ocular globe are difficult to dissect, especially for immunohistochemistry or immunofluorescence [49]. Inside the eyeball, the retina, a structure considered part of the central nervous system, is composed of a high diversity of cells with differential morphologies, neurochemistry, and functions [50]. This neural structure has been considered a simple model of the brain because pathological changes observed in the retina have resembled that of the brain [51]. However, this tissue is extremely fragile and easily deformable; based on this Yang et al. [32] designed a new method to fix the retina, which reduces the timeline (from 2–3 days to 1–2 days) of immunofluorescence protocol, maintaining its cytoarchitecture without breakage, deformation, and/or detachment from the eyeball. This protocol is a modification by the same authors, it was called the “old-glue method” [52]. The current method was tested on mouse eyeballs preserving the cell morphology very well.

#### 2.7.2. Super Glue Method

1. Euthanize animals and collect eyeballs;
2. Rinse in PBS pH 7.4;
3. Mark eyeballs with a sharpie pen (blue color is recommended by the authors);
4. Place eyeballs into a Petri dish, eyecup facing up;
5. Remove excess PBS with a Kimwipe^®^;
6. Place super glue (also known as Krazy Glue or Glue 502) into an Eppendorf microtube;
7. Dip a 10 μL tip coupled to a pipette into a microtube with super glue, remove immediately;
8. Contact the central cornea with the tip and wait for 1 min;
9. Dip the sclera attached to the tip pipette briefly into an Eppendorf microtube containing super glue, remove quickly;
10. Immerse eyeball immediately in PBS for 1–2 s, allowing the super glue to harden;
11. Remove excess PBS with a Kimwipe^®^;
12. Use a syringe needle to poke a hole in the edge of the cornea (it also eliminates pressure and osmotic difference);
13. Carefully remove attached tip-cornea and lens;
14. Submerge eyecup in paraformaldehyde at RT for 30 min;
15. Transfer to 30% sucrose in PBS at RT 30 min;
16. Embed in OCT, preserving sagittal plane of the eyecup parallel to the bottom, ultracold at −80 °C;
17. Obtain 12 μm sections using a cryostat.

#### 2.7.3. Immunofluorescence Method

1. Permeabilize with 5% donkey serum in PBS plus 0.2% Triton X-100;
2. Incubate primary antibody diluted in donkey serum, Triton X-100 in PBS at 4 °C overnight;
3. Secondary antibody diluted in the same solution RT for 1 h;
4. Finally, apply mounting medium anti-fade (Sigma).

#### 2.7.4. Key Points

**Figure 7:**
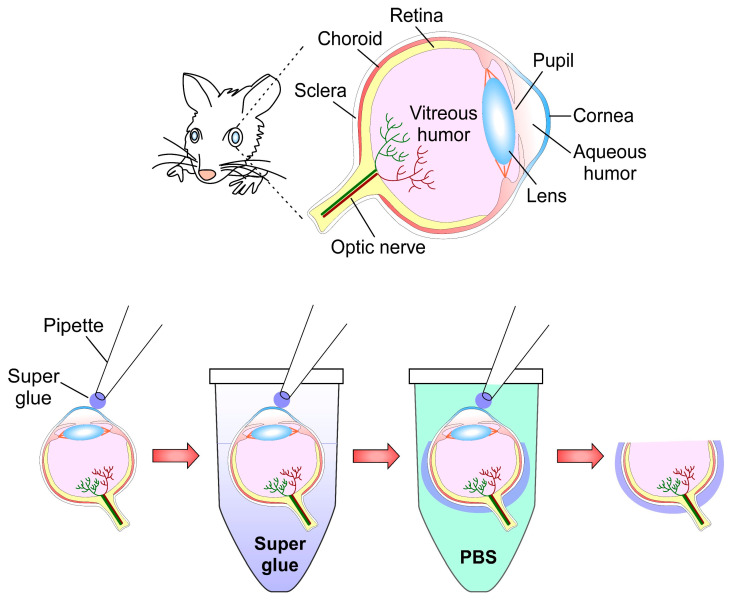
Super glue method for optimal preservation of retina: Schematic representation of mouse ocular globe and its anatomical structures (**upper**). Super glue attached from tip of the pipette to the mouse cornea (**left**), microtube Eppendorf containing super glue until limits of sclera; next, submerge immediately (1–2 s) into PBS-filled Eppendorf and remove excess of PBS. Poke micro-hole in the edge of the cornea, extract complex tip-cornea and lens (**right**), and proceed with fixation step. Figure based on Yang et al. [32].

Consider adding steps 1–14 of super glue method are shown in Figure 7 to your immunofluorescence protocol.

#### 2.7.5. Disadvantages:

(a) You need to look for alternatives to replace super glue if it is not available in your country. Alternatively, you can test glues that harden immediately with water contact;
(b) Super glue is very sticky and can be spread. Attaching a pipet tip to an eyeball requires practice to avoid attaching other things near to the experiment area.

### 2.8. Optimized Fixation Using Ultracold Methanol to Improve Immunofluorescence

#### 2.8.1. Drawbacks to Face

Disadvantages to face that are presented in (Figure 1): 7–9 and 16.

This improvement method for immunolocalization by Hagedorn et al. [28] was designed to counteract drawbacks given by long-term chemical fixation such as: (a) epitope masking, (b) loss of proteins, (c) background, (d) preserving antigenicity, and (e) loss of native structure of proteins, as well as (f) antigen accessibility [53]. To prevent disadvantages given by the fixative solutions, the authors created a technique that reduces antigen mobility by rapidly applying a freezing fixation with methanol at a low temperature. The application of this organic solvent induces membrane permeabilization, avoiding the use of detergents and therefore any subsequent chemical denaturation of proteins and membranes. As a consequence of the use of low temperatures, the authors reported that the ultracold methanol fixation method may cause hexagonal ice crystals inside the cells or organelles, as has been reported previously [54]. Nevertheless, nuclear counterstaining with DAPI can be used to monitor the formation of hexagonal ice, and this label must be an indicator of cell exclusion. The authors reported that ultracold methanol fixation results in excellent permeabilization of tissues and high preservation of antigens. This technique was tested on *Dictyostelum* cells.

#### 2.8.2. Ultracold Methanol Fixation for Immunocytofluorescence

**Figure 8:**
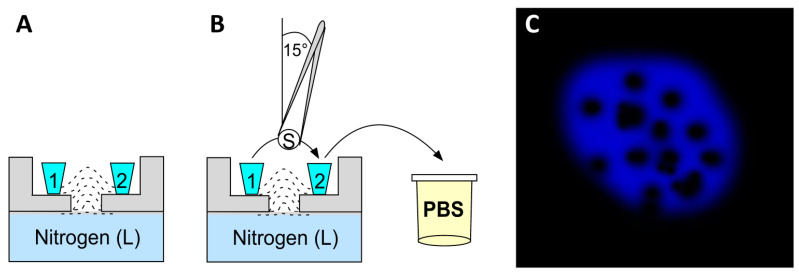
Ultracold fixation method: (**A**) components for cryofixation: bottom to top, source of liquid (L) nitrogen and holder (gray) to put two methanol containers (1 and 2 in cyan); (**B**) tweezers previously submerged in silicone grease holding sapphire coverslips 15° from vertical, plunged in ultracold methanol for a few seconds, transferred to another box with ultracold methanol, unfreeze and monitored at proper temperature to find plasmatic membrane, extracellular proteins, or proteins in the cytoplasm; (**C**) honeycomb pattern of nucleus given by ice crystals and revealed by the DAPI stain. A and B based on Hagedorn et al. [28].

1. Wash cells in fresh growth medium or Sorensen’s buffer (15 mM KH_2_PO_4_, 2 mM Na_2_HPO_4_, pH 6.0);
2. Remove excess liquid (but do not over-dry to avoid cell surface tension);
3. Using tweezers previously submerged in silicone grease take sapphire coverslips with the cell layer facing upwards and wait for a few seconds;
4. Tilt the coverslip with cells at approximately 15° and plunge in ultracold methanol (−85 °C) for a few seconds. Quickly transfer it to another container with ultracold methanol;
5. Unfreeze and monitor the coverslip for 30–60 min until its temperature reaches −45 to −40 °C, for plasma membrane or extracellular proteins; use −35 °C for cytoplasmic proteins;
6. Plunge the coverslip vertically into PBS at RT;
7. Rapidly warm the coverslips and remove excess methanol with gentle movements through the air–buffer interface. Do not move laterally, as this will detach the cell layer. See Figure 8A,B for Steps 4–7;
8. Use cells for immunocytofluorescence or store at −20 °C.

#### 2.8.3. Immunofluorescence Protocol

1. Incubate coverslips for 30 min in blocking buffer (PBS, 0.2% gelatin and 0.1% Triton X-100);
2. Incubate with primary antibody diluted in blocking buffer for 60 min;
3. Wash twice for 5 min with PBS;
4. Incubate in secondary antibody for 60 min at RT;
5. Wash twice for 5 min with PBS;
6. Counterstain nuclei with DAPI;
7. Plunge in water, dry, and mount using Prolong antifade (Molecular Probes), or alternatively use Fluoromount G (Southern Biotechnology Associates);
8. The samples can be stored protected from light for months at 4 °C.

Observations: If cells suffer structural damage due to the formation of ice crystals, a honeycomb pattern of DAPI (see Figure 8C) will be observed when imaging in a confocal microscope. Those cells are not useful for the analysis of results.

#### 2.8.4. Key Points

Consider adding steps 3–8 of ultracold methanol to your immunofluorescence protocol.

#### 2.8.5. Disadvantages

(a) Requires specialized coverslips (made of sapphire);
(b) Rapid-freeze fixation induces the formation of ice crystals, producing cell injury.

### 2.9. A Simple Immunofluorescence Method to Locate Proteins into the Nucleolus

#### 2.9.1. Drawbacks to Face

Disadvantages to face that are presented in (Figure 1): 14 and 15.

**Figure 9:**
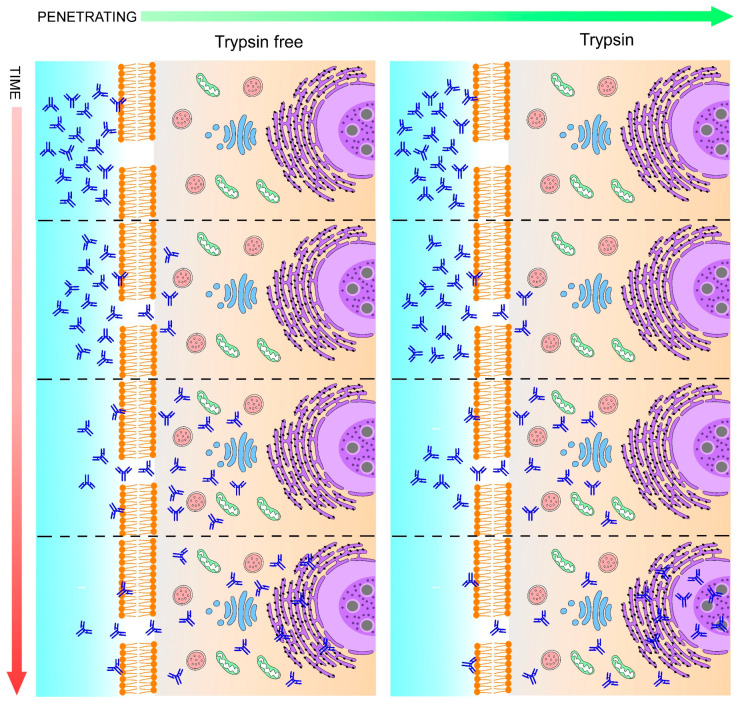
Timeline of antibody penetration: Left, standard immunofluorescence method is not adequate for antibodies to reach deep structures such as the nucleolus and its components, namely the granular, fibrillar center, and dense fibrillar (represented by circles within the nucleolus). Under conventional immunolabeling method range an antibody penetration between 8–9 µm has been reported [43]. Right, deep structures are reached by antibodies after trypsin treatment.

Deep inside eukaryotic cells, more than 4500 proteins have been identified within the nucleolar structure. Nucleolar organization is composed principally of fibrillar components [55]; this fibrillar nature of the nucleolus is conjugated to interfere with the penetration of antibodies and therefore their interaction with epitopes to describe protein distribution (Figure 9). Svistunova et al. [31] designed a simple and rapid method of immunofluorescence to facilitate the accessibility of antibodies into nucleolus, avoiding undesirable effects related with the use of chimeric proteins (such as mislocalization and overexpression). Authors demonstrated that using proteinase treatment allowed immunostaining into the nucleolar structure of HeLa cells.

#### 2.9.2. Fixation

1. Grow cells in monolayers;
2. Fix cells in cold (−20 °C) methanol, 10 min.

#### 2.9.3. Immunofluorescence

1. Membrane permeabilization with 0.5% Triton X-100;
2. Proteinase treatment, 5 min: 50 mg/mL of Proteinase K (Sigma-Aldrich) in PBS at 37 °C. Stop reaction by transferring samples into ice-cold PBS;
3. Wash in PBS;
4. Blocking step in 1% BSA diluted in PBS, 30 min;
5. Primary antibody incubation 60 min at 25 °C;
6. Rinse with 0.1% BSA, 0.5% Tween 20 in PBS;
7. Secondary antibody incubation 45 min at 25 °C;
8. Wash and mount onto slides applying anti-bleaching agent DABCO (Sigma-Aldrich).

#### 2.9.4. Key Points

Consider adding steps 2 of fixation and 2 of immunofluorescence to your immunofluorescence protocol.

#### 2.9.5. Disadvantages

(a) Methanol is more effective in locating proteins inside nucleoli, however, this fixative method modifies cellular morphology;
(b) Proteinase induces protein and antigen damage—authors do not recommend coupling this method with an estimation of proteins by quantification and;
(c) Proteinase treatment is only recommended for high amounts of antigens present in high concentrations within the cell.

### 2.10. Improve Immunofluorescence Combining Heat and Proteolytic-Induced Epitope Retrieval Methods

#### 2.10.1. Drawbacks to Face

Disadvantages to face that are presented in (Figure 1): 7–9, 14 and 15.

**Figure 10:**
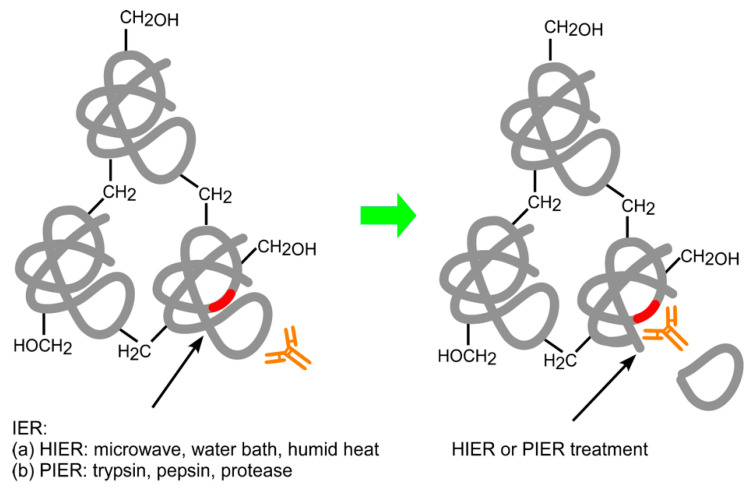
Unmasking antigenic sites by methods to induce epitopes retrieval (IER): Antigenic site (red) is not reachable for antibody binding (**left**) before unmasking treatments. After heat (HIER) or proteolytic (PIER) treatment antigenic sites are available for antibody binding (**right**).

Yabuki et al. [26] applied a combination of heat and proteolytic antigen retrieval methods. One of the most common consequences of the formaldehyde fixation is the crosslinking of methylene bridges between formaldehyde and proteins [56], making it impossible for antibodies to reach the epitopes of proteins, leading to weak specific signals of immunofluorescence staining and favoring the high background given by false-positive staining if an antigen retrieval step is not applied before the immunofluorescence method. HIER (heat-induced epitope retrieval) and PIER (proteolytic-induced epitope retrieval) (Figure 10) are methods that have been designed to break bridges between proteins and aldehydes, resulting in the release of epitopes. Authors provided a combination of HIER and PIER pretreatments to increase specific antibody signals, considering that both methods applied alone could induce non-specific signals. It was tested in renal samples of dogs.

#### 2.10.2. Immunofluorescence Formalin-Fixed Paraffin-Embedded Method

1. Deparaffinize formalin-fixed 3 μm sections;
2. Antigen retrieval treatment: (a) PIER: 0.1% trypsin (Sigma-Aldrich) 30 min at 37 °C, and then (b) HIER: Tris-EDTA buffer pH 9.0, heat into a domestic microwave oven at 750 W (Panasonic). Apply as follows: 5 min of pre-warming, 10 min of heating, and 20 min of cooling at RT;
3. Wash with PBS 10 mM pH 7.4;
4. Blocking buffer (0.25% casein (Sigma Aldrich) in PBS);
5. Primary antibody incubation diluted in blocking buffer 30 min at RT;
6. Wash in PBS;
7. Secondary antibody incubation for 30 min at RT.

#### 2.10.3. Key Points

Consider adding step 2 to your immunofluorescence protocol.

#### 2.10.4. Disadvantages

(a) PIER and HIER could generate tissue morphology or epitope damage. Appropriate optimization of incubation, degrees of heating, times, and concentration of chemical reagents are important factors to consider when you apply it to other fixing and embedding methods, and for each antibody. Consider always performing trypsin digestion before microwave treatment to avoid excessive digestion. Tris-EDTA provided better results than the citrate buffer for HIER.

## 3. Additional Tips

1. Drawback to face (Figure 1): 13. As an alternative to avoid artifacts or undesirable stains given by crosslinking between the animal serum and antibodies of similar species, use a buffer free of animal proteins (e.g., “Blocking Reagent, Animal-Free Blocker (5x)–Vector, SP-5030-250”). This solution can be used for immunohistochemistry and immunofluorescence;
2. For the antigen retrieval of floating and slide-mounted sections, place floating samples in 1.5 mL tubes or cover-mounted samples (slide mounted) in Coplin jars with a citrate buffer pH 6.0 at 80 °C in a beaker or container. Place in a water bath for 20 min. Temperatures of 80 °C and citrate buffer break cross-bridges generated during fixation;
3. Elimination of fluorescent precipitates from fluorochrome-conjugated antibodies: Sometimes old antibodies, or those that are subjected to continuous temperature changes, form precipitates which increase the background signal. Briefly, vortex fluorescent-conjugated antibodies at medium or low velocity for a few seconds, then pulse spin (≈2 s) in a microcentrifuge. Do not invert the tube. Pipette the antibody slightly below the surface of the solution, avoiding the walls of the tube. The precipitates will gather at the bottom of the tube, although the best way to avoid fluorescence precipitates is to aliquot the antibodies when they are totally new;
4. Re-dye nuclei counterstaining after photobleaching.
  - Protect from light and immerse slices in 96% ethanol;
  - Hold slices with fine forceps and carefully remove with a pipette tip the nail varnish of the interface among coverslip and slices;
  - Immerse in fresh ethanol 96% until all nail varnish is removed with the tip, or by using a fine instrument if needed;
  - Wash three times for 5 min each in PBS;
  - Place slices in ethanol 96% 2 min;
  - Wash in absolute ethanol for 3 min (twice). Avoid over-drying;
  - Perform nuclei counterstain one more time;
  - Plunge slices in distilled water or PBS;
  - Mount and seal with nail varnish.
5. Technique for improving penetration of OCT for frozen sections: Immerse tissues in a 1:1 ratio of: OCT and PBS-0.05%-Tween20-0.1%-Triton X-100-sucrose (consider final concentration of sucrose must be 30% of the total volume). Shake at medium velocity on an orbital shaker, then incubate in whole OCT on a shaker for less than 1 h, and finally rapid freeze;
6. Clean slides and coverslips:
  - Submerge in 70% ethanol supplemented with 0.01% Tween 20;
  - Scrub with a fine and clean brush;
  - Immerse in ethanol 96% for a few minutes;
  - Move to fresh ethanol 96% again;
  - Wiggle each briefly;
  - Dry carefully with Kimwipes^®^ (Kimtech), or alternatively use a microwave oven for a few seconds.
7. Drawbacks to face (Figure 1): 17. Intense glare gave by counterstaining. Reduce emission spectra and/or pinhole diameter into your confocal parameters to avoid over emission;
8. Early membranes permeabilization. During transcardially perfusion: add 0.05% Tween20 and 0.1%-Triton X-100 to wash buffer solution (perfusion or serum replacement). This will increase membrane permeability and, due to the low concentration of detergents, will avoid autofluorescence and membrane damage;
9. Drawbacks to face (Figure 1): 3. Transcardial perfusion is a critical step for immunostaining, to minimize hypoxia level and maintain tissue oxygenation during this procedure, apply oxygenated buffer at 37 °C in the washing step (replacement of blood), and it can prevent tissular ischemia;
10. The success of immunofluorescence implies a good selection of antibodies, as well as their specific application [57]. For this reason, we provide a final recommendation of two alternative websites for improving your immunostaining, suggested reagents, recipes, equipment, and a protocol database:
  - IHC World—Life Sciences Products and Services: As the name implies, it is a full alternative for technical advice for basic concepts, methods, techniques, and protocols; additional sources, such as a forum, blog, distributors, academic or technical jobs, histology issues, and a microscopy section are also included. This webpage displays many sections, and in turn each section displays multiple options that are very useful for beginners and experts in immunolabeling (http://www.ihcworld.com, accessed on 23 November 2021);
  - Biocompare, The Buyer’s Guide for Life Scientist: There is a useful database to find antibodies, and this web page includes: antibody suppliers, host species, applications, reactivity, quantity of antibodies, and as very important factors, the number of citations and price are included as well (https://www.biocompare.com//Search-Antibodies/ accessed on 23 November 2021).

## 4. Concluding Remarks

The multifaceted nature of fluorochromes available for immunofluorescence makes it a widely used, valuable, and indispensable technique in the challenging description of the localization, distribution, and quantification of proteins in tissues or cells. Despite that, the wide variety of reagents used for this procedure, as well as the diversity of the biological samples, also causes a great variety of drawbacks—even for a well-established method.

For a long time, the inconsistences of immunolabeling have been the subject of discussion between research groups around the world. Through this review, we described the common drawbacks of the standard method of immunofluorescence to subsequently provide some improvements when the standard method does not allow the appropriate detection of proteins. Facing both scenarios, we want to help the reader in the selection of the best protocol according to their specific needs. It is important to clarify that the advice given in this review does not solve other important issues associated with immunofluorescence, such as antibody specificity tests and establishment of adequate controls, for which there are detailed studies [58,59,60,61,62].

Since there are no similar actualized technical recaps in the literature, throughout a series of 10 protocols, we have sought to show in detail their strengths and weaknesses, as well as the conditions in which each of them is particularly useful in a certain type of organ, tissue, or cell. The methods herein, and the 10 extra tips, offered alternative solutions to overcome drawbacks in protein alterations, metabolic and biochemical changes, sample damages, low permeabilization, low antibody signal, high background signal, unmasking epitopes, as well as the over-emission of fluorescence given by nuclear counterstain.

The so-called key points are to emphasize the technical value of the protocols. They are mainly aimed at experienced researchers who may prefer not to change their immunostaining protocol to avoid facing other variables given by these 10 protocols; as such, this could be a more suitable and adaptable solution to the resources of each laboratory. Furthermore, we propose that a combination of some of these key points could be integrated in a single method, leading to a high-quality immunofluorescence.

For the benefit of the reader, our main objective for this review, in addition to describing improvements in a user-friendly and easy-to-follow approach, is to turn this manuscript into a practical guide to be used directly as an experimental benchmark.

## Figures and Tables

**Table 1:** Panel of the use of terms in immunostaining.

	Enzymes	Fluorochromes
**Prefix**	**Suffix**	
**Immuno**	Immunochemistry (also immunoenzyme)	Immunofluorescence
**Cyto (cell)**	Cytochemistry	Cytofluorescence
**Histo (tissue)**	Histochemistry	Histofluorescence

## Data Availability

Not applicable.

## Supplementary Materials

The following supporting information can be downloaded at: https://www.mdpi.com/article/10.3390/ijms23031426/s1.

## Abbreviations

ASE	Antibody signal enhancer
BS3	Bis(sulfosuccinimidyl)suberate
BSA	Bovine serum albumin
DAPI	4′,6-diamidino-2-phenylindole
EDTA	Ethylenediamine-tetra-acetic acid
GFP	Green fluorescent protein
HEPES	(4-(2-hydroxyethyl)-1-piperazineethanesulfonic acid)
HIER	Heat-induced epitope retrieval
IER	Induced epitope retrieval
MAPK	Mitogen-activated protein kinases
PBS	Phosphate-buffered saline
PBS-NHS	PBS-normal horse serum
PIER	Proteolytic-induced epitope retrieval
RT	Room temperature
TGB	Tris-glycine buffer
