# Supplementary material for: Ten Approaches That Improve Immunostaining: A Review of the Latest Advances for the Optimization of Immunofluorescence
Source: Int J Mol Sci. 2022 Jan 26;23(3):1426. doi: 10.3390/ijms23031426 (PMC8836139; doi:10.3390/ijms23031426)
Supplement: Supplementary file 1 [file ijms-23-01426-s001.zip › ijms-1527223-supplementary.pdf]

Supplementary information

Animals

Male Wistar rats were housed in cage under standard room temperature and light-dark controlled cycles, with access to food and water *ad libitum* and were handled in accordance with the rules for Research in Health Matters (Mexico), and institutional approval of the animal's care committee (RTI21-14) of Institute of Cellular Physiology, UNAM.

Materials and methods

Male Wistar rats (270-300 g) were anesthetized with pentobarbital (50 mg/kg i.p.), transcardially perfused with 250 mL of 0.9% saline solution, followed by 250 mL of 4% paraformaldehyde in PBS pH 7.4. After, the Bicep femoris was harvested and post-fixed in PFA 2% for 24 hours and dehydrated with 30% sucrose in PBS for 24–48 Hr. Muscles were cryoprotected; after, 35 µm symmetric longitudinal sections from the outer side of skeletal muscle were obtained in a cryostat at −20 °C, then were mounted on a slide and maintained at 4 °C. For immunohistochemistry, the slide was manipulated horizontally surrounding the tissue with a hydrophobic barrier. Slides were blocked for 1 hour (in BSA 2%/triton 0.2% or ASE block buffer), and incubated with a 1:100 dilution in BSA 2%/triton 0.2% or ASE antibody incubation buffer of rabbit polyclonal anti S100 β antibody (ab41548, abcam) for 72 hours at 4 °C, followed by incubation with a 1:200 donkey anti rabbit Alexa Fluor 546 antibody (a10040, abcam) and 3 mg/mL αbungarotoxin–Alexa Fluor 488 conjugated (Thermo Fisher, B13422) for 2 hours at room temperature. Finally, the fluorescent mounting medium used was Dako (Gentaur, S302380).

All images were acquired using an inverted microscope Zeiss Axio Observer.Z1 with confocal unit LSM 800, a Plan-Apochromat 63X magnification, 1.4 of numerical aperture and oil immersion objective was used. Laser lines of 488 and 561 nm were applied to obtain fluorescence emission under the Airyscan configuration combined with Z-stack mode.

Fluorescence intensities were quantified using software ZEN 2.3 Blue Edition 2012 (Carl Zeiss, Jena, Germany).
